# Investigation of central pattern generators in the spinal cord of chicken embryos

**DOI:** 10.1007/s00359-024-01694-6

**Published:** 2024-03-23

**Authors:** Cristián Gutiérrez-Ibáñez, Douglas R. Wylie

**Affiliations:** 1 Department of Biological Sciences, University of Alberta, Edmonton, AB T6G 2E0 Canada; 2 Grass Laboratory, Marine Biological Laboratory, Woods Hole, MA USA

**Keywords:** Avian, Locomotion, CPG, Connectivity, Interneuron, Neural circuit

## Abstract

For most quadrupeds, locomotion involves alternating movements of the fore- and hindlimbs. In birds, however, while walking generally involves alternating movements of the legs, to generate lift and thrust, the wings are moved synchronously with each other. Neural circuits in the spinal cord, referred to as central pattern generators (CPGs), are the source of the basic locomotor rhythms and patterns. Given the differences in the patterns of movement of the wings and legs, it is likely that the neuronal components and connectivity of the CPG that coordinates wing movements differ from those that coordinate leg movements. In this study, we used in vitro preparations of embryonic chicken spinal cords (E11–E14) to compare the neural responses of spinal CPGs that control and coordinate wing flapping with those that control alternating leg movements. We found that in response to N-methyl-d-aspartate (NMDA) or a combination of NMDA and serotonin (5-HT), the intact chicken spinal cord produced rhythmic outputs that were synchronous both bilaterally and between the wing and leg segments. Despite this, we found that this rhythmic output was disrupted by an antagonist of glycine receptors in the lumbosacral (legs), but not the brachial (wing) segments. Thus, our results provide evidence of differences between CPGs that control the wings and legs in the spinal cord of birds.

## Introduction

Central pattern generators (CPGs) are assemblies of neurons that orchestrate rhythmic patterns of neural activity without rhythmic input (Stein et al. 2015; Katz 2016). In vertebrates, CPGs for locomotion are found in the spinal cord and consist of several cell types, including motor neurons and both excitatory and inhibitory interneurons (Catela et al. 2015; Gosgnach et al. 2017). In vertebrates, the organization of spinal CPGs and the function of their different components have been studied in detail in relation to swimming and walking. Surprisingly, less attention has been given to neural circuits related to the motor control of flight, a major innovation in locomotion among vertebrates. Powered flight has evolved, independently, twice in extant vertebrates, in bats and birds. In both cases, the forelimbs have evolved into wings, which move up and down in coordination to generate lift and thrust (Norberg 2012). The pattern of muscle activation necessary for flying differs significantly from that of other locomotion types. Most notably, in both swimming and walking, locomotion entails alternating motor rhythms of the left and right sides of the body, while in flying, the beating of both wings occurs synchronously (Norberg 2012). For swimming and walking, spinal CPGs coordinate the alternation of both the body halves and of intralimbic flexor and extensor muscles, in the case of walking (Butt et al. 2002; Catela et al. 2015). This coordination is achieved in all vertebrates through a small and highly conserved pool of cardinal spinal interneurons (Gosgnach et al. 2017). The ability of CPGs to generate different motor outputs in different species is linked to variations in the diversity of these interneurons and their pattern of connectivity (Katz and Harris-Warrick 1999; Katz 2016; Gosgnach et al. 2017). While birds have evolved the ability to fly, they have, for the most part, conserved the bipedal walking of their ancestors. Thus, the spinal cord of birds is likely to harbor at least two types of CPGs that generate two different motor patterns: wing beating with forelimbs and walking with hind limbs. Given this, one would expect that the neuronal components and connectivity of a putative CPG that coordinates wing movements in birds would differ greatly from those that coordinate other types of locomotion, including hind limb walking in birds (but see Pocratsky et al. 2020 for alternatives on how coordination could be archived). A recent study (Haimson et al. 2021) has provided evidence to support that the circuit of the CPG that controls wing motions is different from the one that controls leg movements in birds. In birds, ephrin-B3, a spinal midline protein, that in mammals instructs the wiring that enables limb alternation, lacks several motifs present in other vertebrates, diminishing its affinity for the ephrin-A4 receptor. This results in a larger number of excitatory interneurons crossing the midline in the brachial but not in the lumbosacral spinal cord of chicks. Concordantly, ephrin-B3 and ephA4 null mice also show a larger number of excitatory interneurons crossing the midline and a rabbit-like hopping gait (Akay et al. 2006; Restrepo et al. 2011; Borgius et al. 2014). In mice, it has been proposed that the control of alternating left and right motor outputs depends on the balance between inhibitory and excitatory inputs from the contralateral side (Butt et al. 2002; Restrepo et al. 2011; Talpalar et al. 2013). While the coordination of wing beating in birds seems to be driven by an increase in excitatory inputs to the contralateral side, in ephrin-null mice, there are not only additional excitatory inputs but also diminished inhibitory inputs (Borgius et al. 2014). The role of inhibitory neurotransmitters, particularly glycine, in the control of left/right alternation is well established. Blocking glycine receptors produces coordinated left–right motor outputs in a variety of vertebrates (Droge and Tao 1993; Hagevik and McClellan 1994; Kremer and Lev-Tov 1997). This effect is mediated by a particular class of glycinergic commissural interneurons found in all vertebrates, V0 interneurons (Gosgnach et al. 2017). In mice, the genetic ablation of V0 interneurons, 70% of which are glycinergic, results in coordinated left/right motor outputs in vitro and a rabbit-like hopping phenotype (Talpalar et al. 2013). Similarly, in Netrin-1 and the netrin-1 receptor DCC (deleted in Colorectal Cancer) null mice, there is a reduced number of commissural projections from V0 interneurons, and these mice also show synchronous motor outputs (Rabe et al. 2009; Rabe Bernhardt et al. 2012). Therefore, it is possible that the CPG that controls wing beating in birds not only presents increased excitatory inputs, but also alterations to inhibitory commissural circuits, particularly glycinergic ones.

A common approach in the study of spinal circuits in vertebrates, including chickens, is in vitro preparations, where a pattern of rhythmic activity that mimics that of locomotion (fictive locomotion) can be induced pharmacologically, or even occur spontaneously (Kiehn 2006). In chickens, in vitro preparations of the embryonic spinal cord (up to E14) show spontaneous and rhythmic motor outputs, with alternating activity between antagonistic muscles (Barry and O’Donovan 1987; O’Donovan et al. 1992; Chub et al. 1998). In Chick in vitro preparations, as early as E7 (hatching typically occurs on E21) spontaneous motor burst can be recorded or triggered by stimulation of the brainstem, and by E11, alternating burst can be recorded from antagonistic muscles or the corresponding nerve (O’Donovan and Landmesser 1987; O’Donovan et al. 1992). At these developmental stages, motor activity can also be induced in vitro by bath application of N-methyl-d-aspartate (NMDA) and inhibited by NMDA receptor antagonists and glycine receptor agonists (Barry and O’Donovan 1987). Previous studies in the chicken embryonic spinal cord have also shown that, like in other vertebrates (Reviewed in Kiehn and Kjaerulff 1998) each segment is capable of generating rhythmic outputs and that, at least in the lumbosacral spinal cord, motoneuron activity is synchronized along the rostro caudal axis, and mediated through propriospinal projections (Ho and O’Donovan 1993). However, little is known about the coordination of left and right activity in chick spinal cord and if brachial and lumbosacral segments differ in their motor outputs. In this study, we used in vitro preparations of embryonic chicken spinal cords (E11-E14) to compare the neural responses of spinal CPGs that control and coordinate wing flapping with those that control alternating leg movements.

## Methods

All experiments were approved and performed in accordance with the Marine Biological Laboratory (MBL) Animal Care and Use Committee (IACUC) protocol 19-08G/GF/Gutierrez. Fertilized chicken eggs were obtained from a commercial supplier (Charles River Laboratories, Inc.). Before incubation, the eggs were maintained at 14 °C for up to 10 days. The eggs were incubated at 39 °C and 50% humidity until they reached E11–E14. Motor activity was recorded between E11 and E14 using isolated spinal cord preparation. Embryos were extracted from the eggs, quickly decapitated, eviscerated, and then transferred to cold (4 °C), oxygenated, sucrose-substituted Krebs solution (210 mM sucrose, 3 mM KCl, 3 mM MgCl2-6H2O, 23 mM NaHCO3, 1.2 mM NaH2PO4-6H2O, 11 mMD1-glucose) in a sylgard-lined petri dish. A ventral laminectomy was performed under a dissection microscope, and the main nerve plexus enervating the limbs were exposed and cleared of muscle and connective tissue. After dissection, the spinal cord was transferred to a recording chamber lined with sylgard, where it was secured using entomology pins. The chamber was perfused (15–20 ml per minute) with artificial cerebrospinal fluid (aCSF) solution (144.2 mM Na^+^, 129 Cl^−^ 3 mM K^+^, 1 mM Mg^2+^, 23 mM HCO_3_, 1.2 mM Na_2_HPO_4_H2O, 2 mM Ca, 30.53 mM glucose) at 28–30 °C, that was bubbled continuously with Carbogen (95% oxygen, 5% CO_2_). The tissue was left to rest for at least 30 min before recording or adding pharmacological agents.

**Fig. 1:**
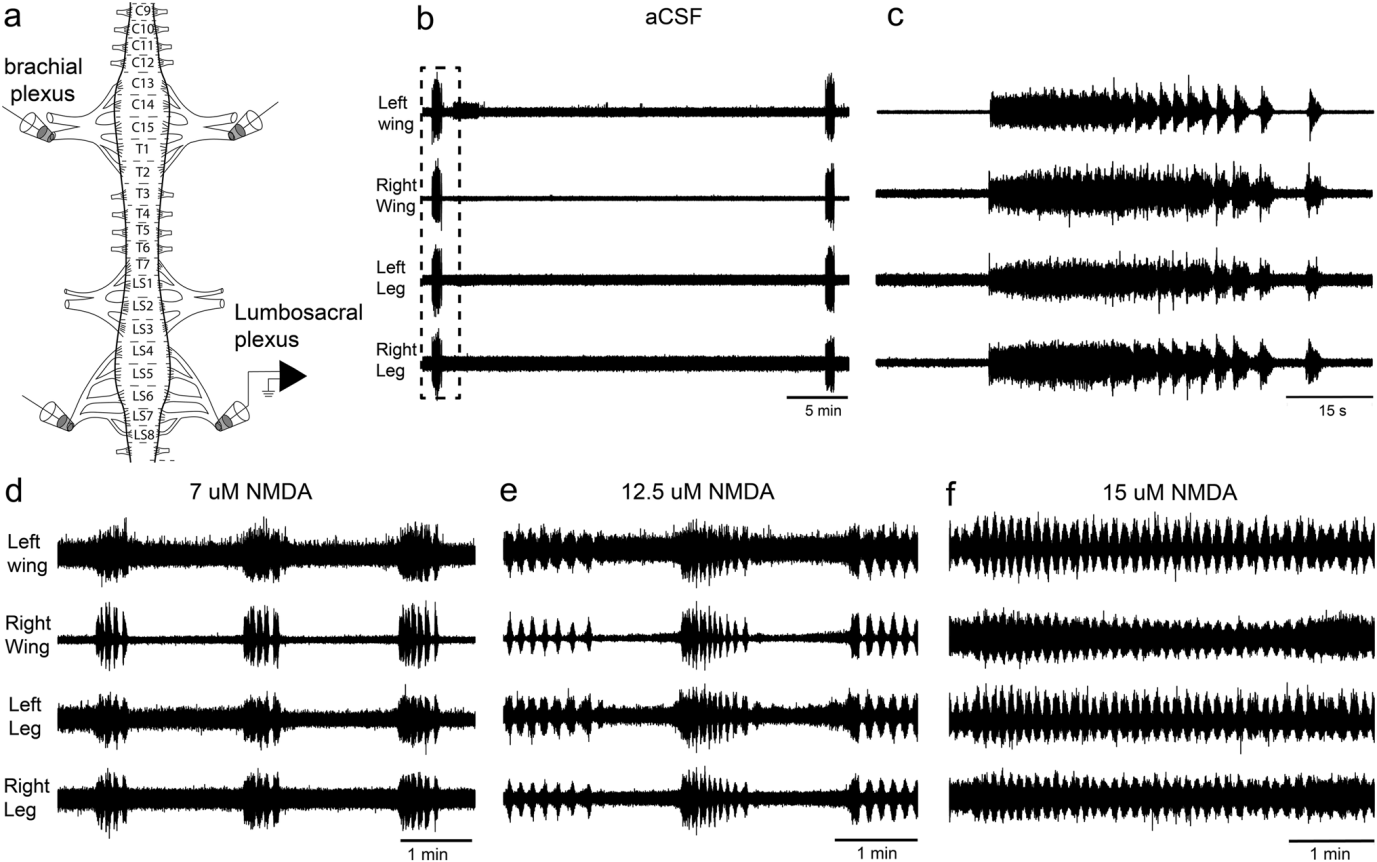
Spontaneous and NMDA induced rhythmic motor activity in the chick spinal cord. **a** A schematic of the experimental setup. Suction electrodes were used to record activity from nerve roots bilaterally in the brachial and lumbosacral spinal cord of E11–E14 chick embryos in vitro. **b** Examples of spontaneous bursts of activity in the chick’s spinal cord. **c** The details of a burst that occurred only once per hour. Spontaneous bursts were approximately 1 min in duration and started with tonic activity that transitioned to discrete bursts. **d** The addition of a low concentration of NMDA (7 μM) results in a higher frequency of bursts similar to those generated spontaneously (**b**, **c**). **e** Shows that at higher concentrations of NMDA in the bath continuous bursts appear, intermingled with longer duration bursts similar to the spontaneous bursts. **f** At a concentration of 15 μM of NMDA (or higher) burst activity becomes continuous with no tonic activity

Recordings were obtained, using glass electrodes with fire-polished tips of between 100 and 300 μm, attached to a shielded suction electrode (AM-systems, cat. no. N:573040), from the nerve stumps of the legs or wings. Figure 1a shows a schematic of the chick spinal cord and nerve plexus in the brachial and lumbosacral areas. In the brachial area, dorsal and ventral roots from segments C13 to T2, which contain the motor pools of the wing (Hollyday and Jacobson 1990), fuse together into a large nerve plexus that then splits into two major branches, an inferior and a superior. Each of these branches then splits into branches that innervate the individual muscles (both extensors and flexors) of the wing and thorax (Phelan and Hollyday 1990). Recordings of the brachial motor activity from either the major plexus stomp before it splits into the main branches or one of the main branches. In the case of the lumbosacral area, motor pools for leg muscles arise from segments T7 to LS8, and give rise to two mayor plexuses, an anterior and a posterior one (Landmesser and Morris 1975). The anterior plexus receives innervation from segment T7 to LS3, and similar to the brachial plexus, nerves (dorsal and ventral roots) from these four segments fuse together and then split into individual nerves. In this case it gives rise to several nerves, including the sartorious and femotoborialis nerves (Landmesser and Morris 1975; Ho and O’Donovan 1993). The most posterior plexus receives innervation from segments LS4–LS8, which contain the motor pools of many flexor and extensor muscles of the leg (Hollyday 1980). The nerves (dorsal and ventral roots) of these segments fuse into the sciatic nerve. Motor activity in the lumbosacral region was recorded from this large nerve stump, unless otherwise indicated.

Electroneurogram (ENG) recordings were conducted using a differential AC amplifier (A-M Systems, model 1700) with a band-pass filter (300 Hz and 3 kHz) and a sampling rate of 5 kHz. Analog signals were digitized using a CED Power 1401 (CED Ltd, UK) and recorded on a PC running Spike 2 software (v8.17, CED). Rhythmic locomotor activity was induced by bath application of N-methyl-d-aspartate (NMDA) alone or NMDA combined with different concentrations of hydroxytryptophan (5-HT). Other pharmacological agents used include strychnine, dopamine, picrotoxin, and gabazine (Trocris). These were dissolved in aCSF and bath applied. All solutions containing dopamine were supplemented with l (+)-ascorbic acid (20–30 μM; Merck) to prevent oxidation.

### Data analysis

All analyses were performed using the custom Python code. Analyses were always performed on 200 s samples with steady rhythmic output. The data were rectified and smoothed with a time constant between 0.2 and 0.4 s. The burst start and end were detected automatically in the rectified and smoothed data using a set threshold of 15% of the maximum amplitude. The burst duration was defined as the interval between the onset and offset of a burst. The lag between the two traces was calculated as the difference in phase between the start of a burst cycle. Cross-correlograms were obtained by calculating the Pearson correlation coefficient between one trace and the lagged version of the second tracer over a range of lag times (30 s). Cross-correlograms that start with correlations close to one and then become lower indicate that the traces are in phase. Similarly, traces with cross correlograms that start at − 1 are completely antiphase. Statistical test where performed in R (R Core Team 2022).

## Results

In total, we recorded rhythmic outputs in 29 in vitro preparations of chick spinal cord. In spinal cord of chicks between E11-E14, bursting, rhythmic activity could be recorded from nerve roots either spontaneously (Fig. 1b, c) or by addition of different (5–30 μM) concentrations of NMDA in the bath (Fig. 1d–f). Spontaneous activity could only be recorded after 3–4 h in the aCSF. This activity was sparse, with bursting trains occurring approximately once per hour. These bursts were approximately 1 min in duration and started as tonic activity, which then turned into discrete bursts (Fig. 1c). The bursts were completely synchronous across the left and right sides and between the brachial (wings) and lumbosacral (legs) spinal cords (Fig. 1b). Low concentrations of NMDA in the bath (5–10 μM, n = 3) produced bursts similar to those produced spontaneously, but at increasing frequencies (Fig. 1d). At concentrations over 10 μM of NMDA, periods of constant bursting appeared, intermitted by burst trains similar to those produced at low NMDA concentrations (Fig. 1e). Above 15 μM of NMDA, bursting became continuous, but with some periods of increasing and decreasing amplitudes (Fig. 1f). As with the spontaneous bursts, the activity was always synchronous across the left and right sides and between the brachial and lumbosacral nerves (Fig. 1d–f).

**Fig. 2:**
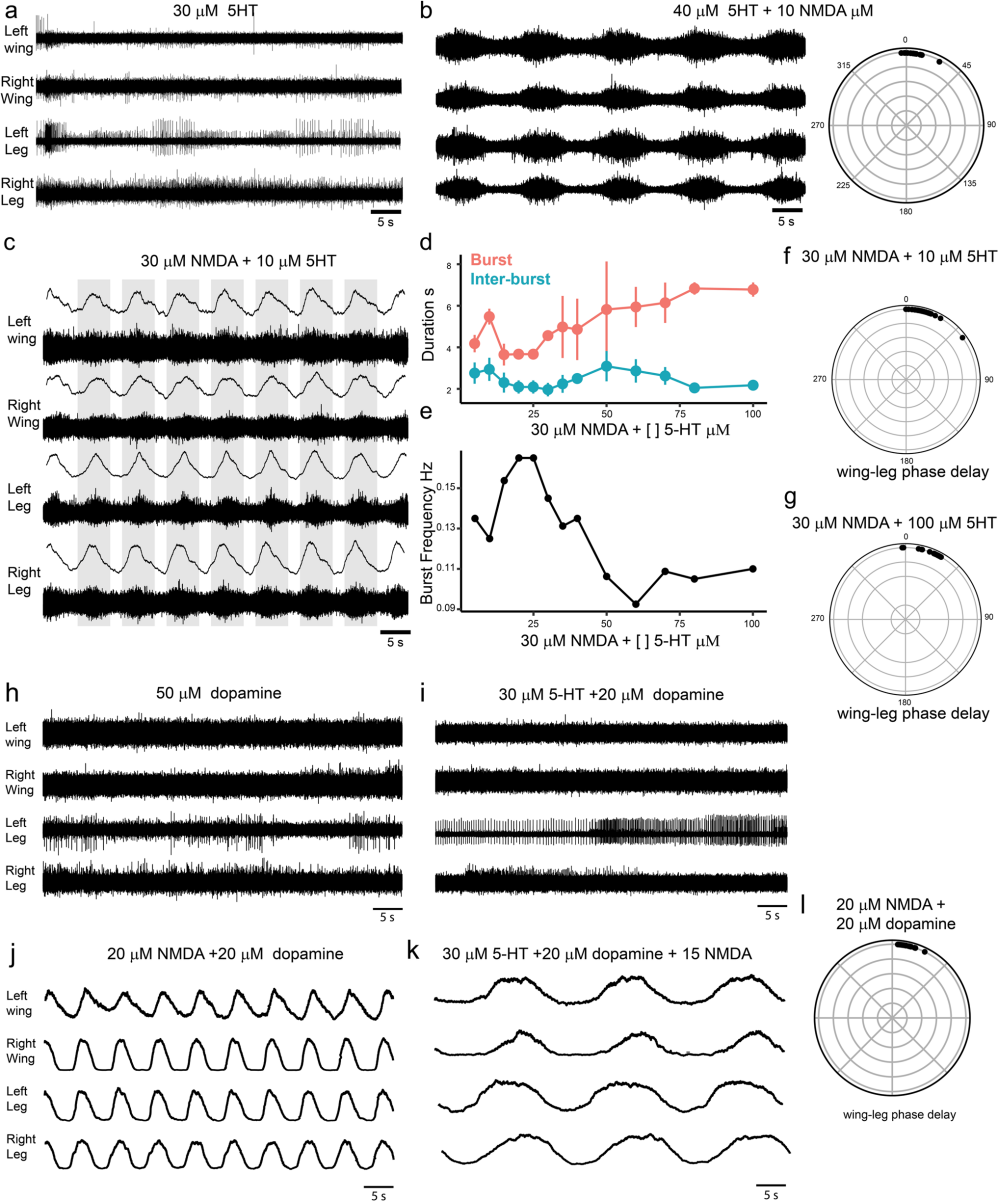
Effects of 5-hydroxytryptamine (5-HT) and dopamine on the rhythmic output of the chick spinal cord.** a** 5-HT (5–50 μM, see Results) alone does not results in a rhythmic output, but addition of a small concentration of NMDA (**b**, 10 μM) results in a regular rhythmic pattern. **c** Shows the regular rhythmic motor output produced by addition of 30 μM of NMDA and 10 μM of 5-HT. The bottom trace shows raw recordings. The top traces show the rectified and smoothed trace. **d** The effect of variation of 5-HT concentration in the bath on the duration of the bursts and inter-burst periods when the concentration of NMDA is held constant (30 μM). **e** The variation in frequency for the same condition as in **d**. **f**, **g** Show the phase lag between the left wing and the left leg for two different concentrations of 5-HT in the experiment shown in d-e. At all 5-HT concentrations. The wing motor outputs showed a delay with respect to those of the legs. **h** Neither dopamine alone (5–50 μM, see results) nor combined with 5-HT (**i**), results in a rhythmic output, but the addition of a small concentration of NMDA (**i–k**, 15 μM) results in a regular rhythmic pattern

Next, we tested the effects of other neurotransmitters on the production of rhythmic motor outputs in the chicken embryonic spinal cord. Serotonin (5-HT) alone did not produce rhythmic outputs (Fig. 2a, 5–50 μM, n = 2). High concentrations of 5-HT (20–50 μM) combined with 10 μM of NMDA produced a slow rhythmic activity with long bursts (Fig. 2b, n = 2) that was also always synchronous across the left and right sides, and between the wings and the legs (Fig. 2b). Higher concentrations of NMDA (20–30 μM) combined with low concentrations of 5-HT (5–15 μM, n = 3) produced stable rhythmic outputs, with constant amplitude (Fig. 2c). Increasing the concentration of 5-HT while keeping the NMDA concentration constant (30 μM) resulted in variations in the duration of bursts, but not of the inter-burst periods (Fig. 2d, n = 3). This resulted in a decrease in the burst frequency (Fig. 2e). The application of 5-HT at any concentration did not change the coordinated rhythmic activity across either the left or right sides or between the legs and wings (Fig. 2f, g).

Dopamine alone did not produce rhythmic activity (Fig. 2h, 10–100 μM, n = 2). Combinations of different concentrations of dopamine and 5-HT did not induce rhythmic activity (Fig. 2i, dopamine = 10–100 μM, 5-HT = 5–40 μM, n = 2). However, increasing the concentration of dopamine (10–100 μM) with a constant concentration of NMDA (20 μM), produced regular rhythmic outputs (Fig. 2j, n = 2). We also tested several combinations of the 3 drugs (5-HT = 10–20 μM, NMA = 7.5–20 μM, dopamine = 50–100 μM, n = 2), which produced rhythmic outputs but all of the combinations produced coordinated burst across the left and right sides as well as between the wing and leg activity (Fig. 2k-i).

**Fig. 3:**
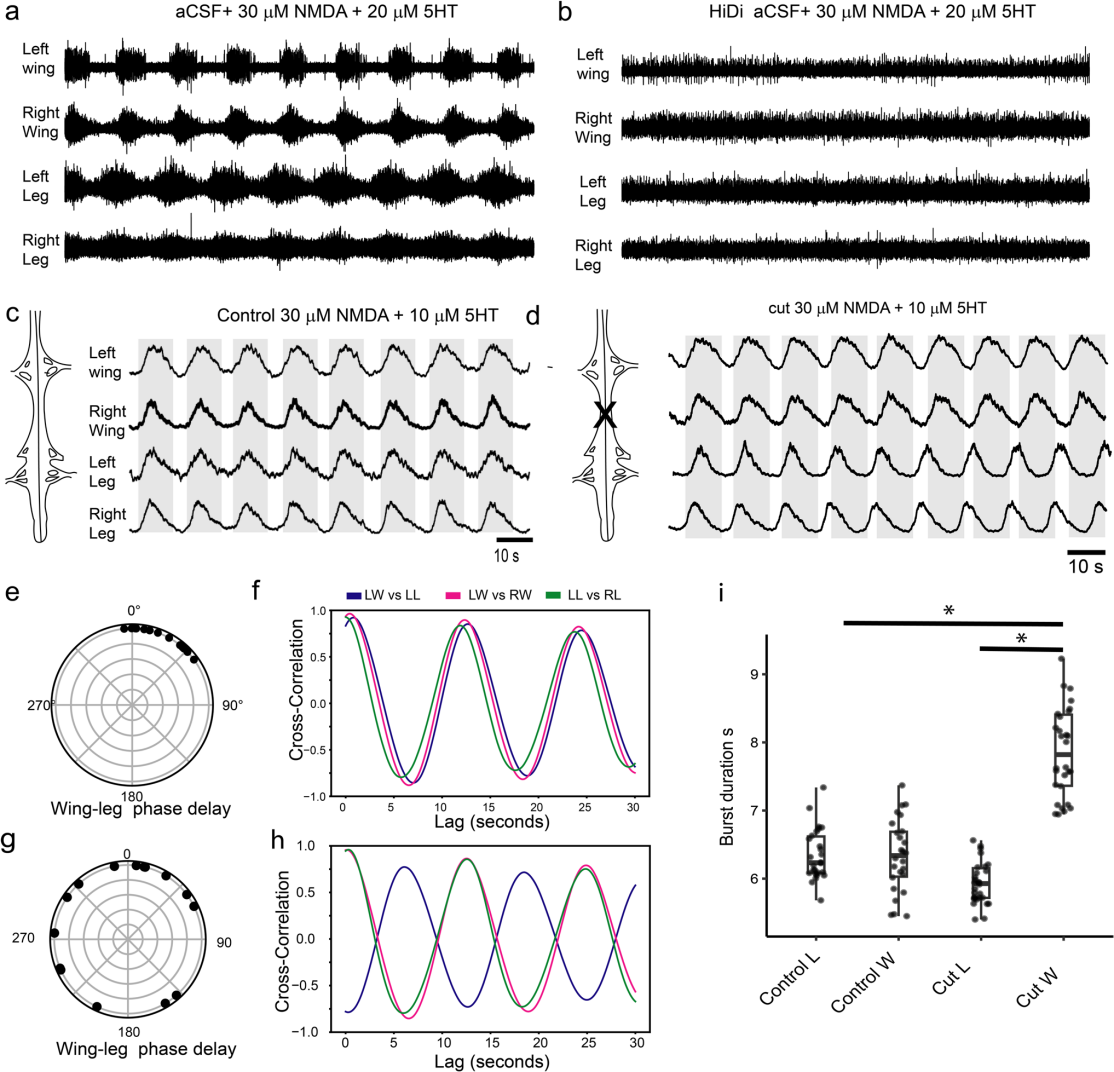
Polysynaptic drive and segmental coordination in the chick spinal cord. **a** A regular rhythmic motor output in the presence of 30 μM of NMDA and 20 μM of 5-HT. **b** Blocking polysynaptic transmission with an artificial cerebrospinal fluid with a high concentration of divalent cations (HiDi, Ca^2+^ and Mg^2+^) suppresses a rhythmic motor output. **c** A regular motor output in the presence of NMDA and 5-HT in a spinal cord were the connections between the brachial and lumbosacral segments are intact. **d** The output in the same spinal cord after the spinal cord has been transected at the mid-thoracic level. **e** The phase lag between the rhythmic output of the legs and wings in the intact spinal cord as shown in **a**. **f** A cross-correlogram between the left leg and the left wing (blue), the left wing and the right wing (magenta) and the left leg and the right leg (green) for an intact spinal cord. **g** and **h** The same as **e** and **f** but for the spinal cord that was transected at the mid-thoracic level. **i** A boxplot of the burst duration for rhythmic motor output of the legs and wings in the intact (control) and cut spinal cord preparations over 200 s. The centerline shows the median. Box limits indicate the range of the central 50% of the data, lines represent the upper and bottom 25% percentiles. Asterisks indicate significant differences

Given the coordination between the brachial and lumbosacral spinal cord we next set to test if the rhythmic output was localized to different sections of the spinal cord and if the rhythmic output depended on mono- or polysynaptic inputs. For the latter, we used aCSF with a high concentration of divalent cations (Hi-Di, 4 mM Ca^2+^, and 2 mM Mg^2+^), which have been shown to suppress polysynaptic transmission (Cazalets et al. 1995, 1996). Hi-Di aCSF resulted in the complete suppression of bursting activity, although some firing was still present (Fig. 3a, b, n = 3). This was true at different concentration of drugs (NMDA = 5–30 μM, 5-HT = 5–15 μM), suggesting that the rhythmic output of the embryonic chick spinal cord is dependent on polysynaptic inputs. Next, we tested if the brachial or lumbosacral spinal cord could produce rhythmic outputs on their own or if one was dependent upon the other. For this we first recorded stable rhythmic outputs from the wing and leg nerves in an intact spinal cord (Fig. 3c) and then from the same spinal cord after it was completely cut between the brachial and lumbosacral portions (Fig. 3d, n = 3). In the intact spinal cord, the wing and legs fire in synchrony (Fig. 3e, f), but when the connection between the two are cut, wing and leg motor outputs fall out of synchrony while still both maintain a left and right coordination (Fig. 3g, h). Bust duration between the two parts of the spinal cord were significantly different after the cut (Fig. 3i, ANOVA, F_3,108_ = 22.47, p < 0.00001), with wing burst duration significantly longer than the burst duration in legs of the cut preparation (Tukey post-hoc, p < 0.001) and the burst duration observed in both the wings and legs of the intact spinal cord preparation (p < 0.001). In other words, in the absence of connections, the brachial spinal cord produced a slower rhythm than the lumbosacral spinal cord under the same conditions.

**Fig. 4:**
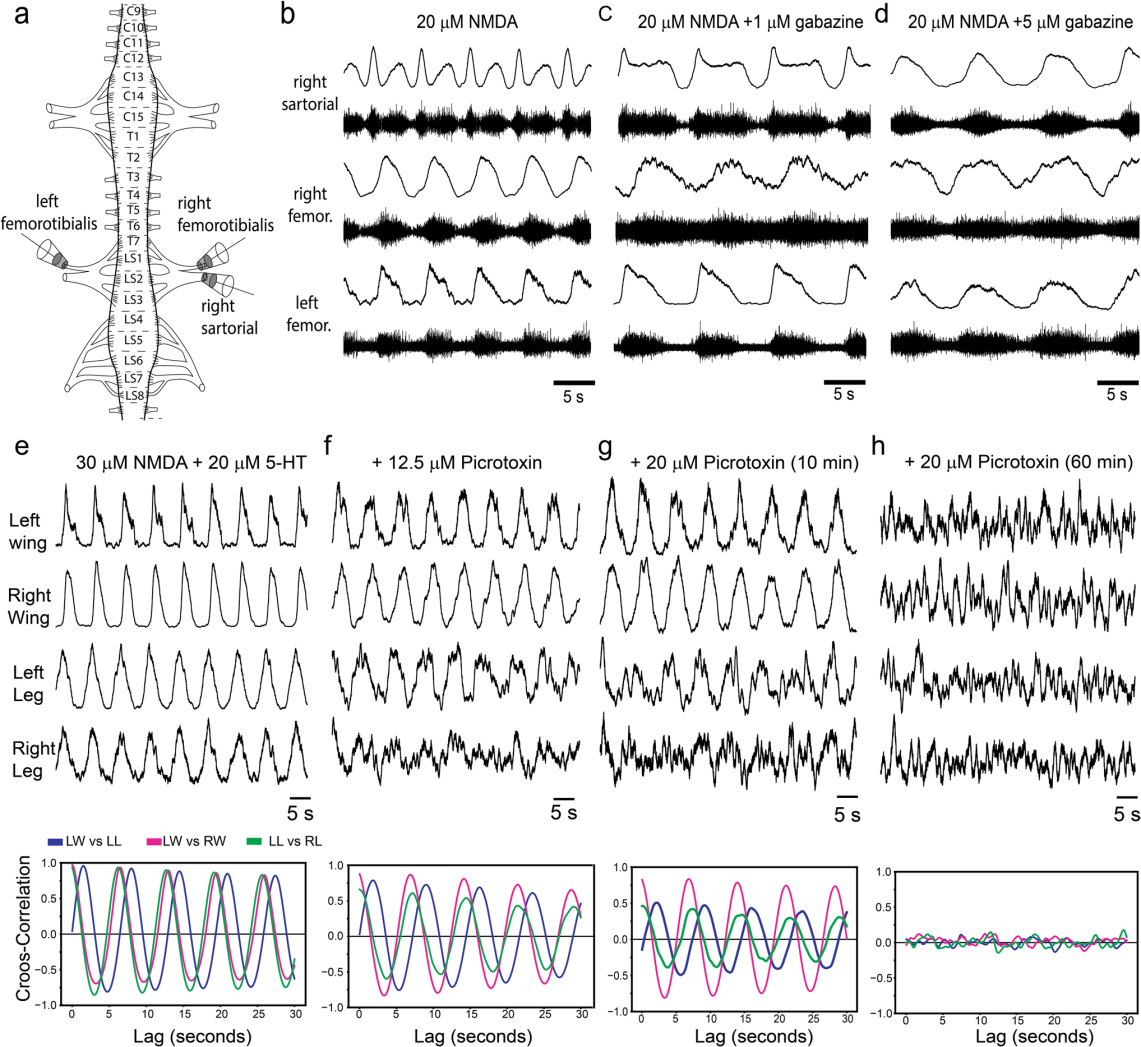
Role of inhibitory neurotransmitters in the rhythmic motor output of the chick spinal cord.** a** A diagram of the recording set-up. In this experiment, two electrodes were attached to the femorotibialis nerves bilaterally, and one to the right sartorial nerve. **b** Raw traces (bottom) and rectified and smoothed traces of the rhythmic motor output in the set-up showed in a, when 20 μM of NMDA was added to the bath. **c** The rhythmic motor output in the presence of 20 μM of NMDA and 1 μM of gabazine, a GABA_a_ receptor antagonist. **d** The same as **c** but with 5 μM of gabazine. **e** Bilateral recording from the wing and leg segments in the presence of NMDA and 5-HT. The bottom panel shows a shows a cross correlogram between the left leg and the left wing (blue), the left wing and the right wing (magenta) and the left leg and the right leg (green) under the same conditions. **f** The same as in **e** but when 12 μM of picrotoxin, a non-selective GABAa receptor antagonist was added. **g**, **h** The same as in **e** and **f** but 10 and 60 min after 20 μM of picrotoxin was added, respectively

**Fig. 5:**
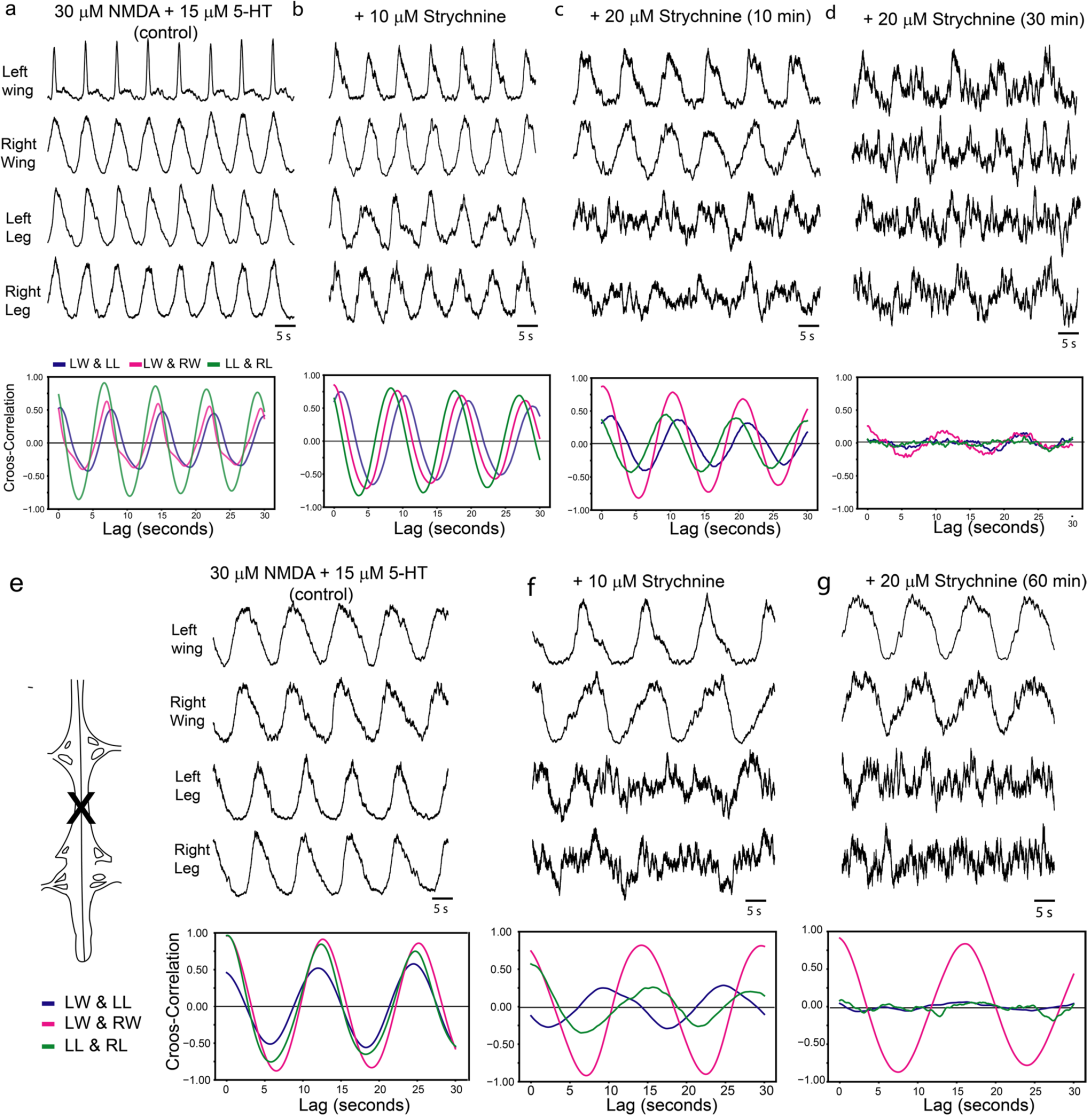
Brachial and lumbosacral segments of the chick spinal cord respond differentially to a glycine receptors blocker. **a** Rectified and smoothed bilateral recordings from the wing and leg segments in the presence of NMDA and 5-HT. The bottom panel shows a cross-correlogram between the left leg and the left wing (blue), the left wing and the right wing (magenta) and the left leg and the right leg (green) under the same conditions. **b** The same preparation as in a but after 10 μM of strychnine, a glycine receptor antagonist, was added to the bath. **c** Recordings from the same preparations as in b but10 min after 20 μM of strychnine was added to the bath. **d** The same preparation as in **c** but 60 min after the strychnine was added. **e** The same as in **a** but after the spinal cord that was cut at the thoracic levels. **f** The same preparation as in **e** but when 10 μM of strychnine, was added. **g** The same as in **e** and **f** but 60 min after 20 μM of strychnine was added

Next, we tested the role of inhibitory neurotransmitters in coordinating the rhythmic activity in the chick spinal cord. With the application of gabazine, a selective GABAa receptor blocker (Ueno et al. 1997), the synchronous activity across the left and right sides and between the legs and wings was not altered (5–50 μM, n = 2, data not shown). In contrast, when we recorded from nerves that innervate antagonist muscle groups in the lumbosacral spinal cord, namely the femorotibial and sartorial nerves (Fig. 4a, n = 2,), we found that in the presence of NMDA alone, the activity of these two nerves on the same side produced an alternating bursting pattern (Fig. 4b). The activity in the femorotibial nerve was synchronous with the contralateral side (Fig. 4b). In the presence of gabazine (1–10 μM) the bursts in the femorotibial and sartorial nerves became progressively more synchronous (Fig. 4c, d), suggesting that the alternation between antagonist groups is mediated by GABAa receptors. Next, we tested the effects of picrotoxin (n = 2, 2.5–20 μM) a less selective GABA antagonist (Takeuchi and Takeuchi 1969). Picrotoxin had no effect on the synchronous coordination across left and right sides or between the brachial and lumbosacral spinal cords (Fig. 4e–h, n = 2), but at higher concentrations of picrotoxin (12 μM) the activity of the lumbosacral spinal cord became more less regular, while the brachial activity was still highly rhythmic and correlated across the two sides (Fig. 4f). This effect was stronger after 10 min of exposure to 20 μM of picrotoxin (Fig. 4g) but after 1 h both the brachial and lumbosacral outputs became arhythmic (Fig. 4g). We also tested the effect of strychnine (n = 3, 2–20 μM), a glycine receptor blocker (Curtis et al. 1971). In the intact spinal cord, strychnine had effects similar to those of picrotoxin, where 10 μM of strychnine (in the presence of 30 μM of NMDA and 15 μM 5-HT), produced a degradation of the rhythmic activity of the lumbosacral but not the brachial spinal cord (Fig. 5a, b). Similar to prototoxin, 20 μM of strychnine produced, initially (10 min), further arrhythmia of the lumbosacral activity but not the brachial activity (Fig. 5c), but after a long exposure (60 min) all activities were arhythmic (Fig. 5d). Next, we tested the effects of strychnine in a spinal cord that had been transected between the brachial and lumbosacral sections (Fig. 5e, n = 2). In this preparation, and as in the intact spinal cord, strychnine disrupted the coordinated rhythm in the lumbosacral regions, but unlike the intact spinal cord, high concentrations of strychnine, for extended periods of time, had no effects on the brachial activities, which remained correlated and rhythmic (Fig. 5f, g).

## Discussion

Here we have shown that, under a variety of conditions, the chick spinal cord produces, in vitro, rhythmic outputs that are coordinated bilaterally and between the wing and leg segments. This coordination occurs spontaneously and under a variety of agonist and antagonists, including NMDA, 5-HT and dopamine (Figs. 1, 2). Previous studies in the in vitro chick spinal cord have shown that rhythmic activity is coordinated between different segments of the lumbosacral spinal cord, and between the brachial and lumbosacral segments (Ho and O’Donovan 1993), but to the best of our knowledge, there has been no study looking at the bilateral coordination in in vitro preparations. Most previous studies in the chick spinal cord have focused on the spontaneous activity generated at early stages and the role this has in the development of CPGs (e.g. O’Donovan and Landmesser 1987; O’Donovan et al. 1992; O’Donovan 1999). Interestingly, we found that serotonin (5-HT) alone is not capable of initiating a rhythmic motor output in the chick spinal cord but does modulates the rhythmic pattern (Fig. 2). This is similar to what occurs in lamprey in vitro spinal cord preparations, where fictive locomotion cannot be initiated by serotonin alone but also acts a modulator of the pattern in the presence of NMDA (Harris-Warrick and Cohen 1985). In contrast, in neonatal mice and rats, serotonin alone is capable of initiating rhythmic locomotor activity in vitro, including alternation between left and right sides (Cazalets et al. 1992; Beato et al. 1997; Branchereau et al. 2000). Serotoninergic projections from the brainstem to the spinal cord have a well-established role in modulating locomotion in all vertebrates (Harris-Warrick and Cohen 1985; McDearmid et al. 1997; Schmidt and Jordan 2000; Gabriel et al. 2009; Flaive et al. 2020). In mammals, serotonin can initiate locomotion in neonates but not in adults (reviewed in Sławińska and Jordan 2019). It is possible that the differences in the role of serotonin in initiating locomotion are related to the different developmental modes of rodents and chickens. Chickens are precocial and can walk almost immediately after hatching, while rats, for example, are altricial and only start walking 12–13 days after birth (Muir 2000). Thus, it is possible that in precocial birds, there is no developmental change in the role of serotonin in modulating locomotion.

### Differences between brachial and lumbosacral locomotor output

Despite finding bilateral coordination in the brachial and lumbosacral segments, we also found that even at early embryonic stages (E11–E14), the lumbosacral (legs) and the brachial (wing) segments show some differences in their locomotor output in response to the same pharmacological treatment (e.g. Figs. 3i, 4e–g, 5). This provides support to the idea that in the avian spinal cord the circuits of the CPGs that produce the motor outputs of wigs and legs are wired differently to produce distinct motor outputs; coordination with the wings and alternations with the legs (Gatesy and Dial 1996). The first evidence in this regard comes from early transplantation experiments in chick embryos, where the brachial segments were replaced by lumbosacral segments, which resulted in alternating movements of the wings (Straznicky 1963). In a similar experiment, transplantation of brachial segments to the lumbar cord results in synchronous leg movements (Narayanan and Hamburger 1971). Recently, Haimson et al., (2021) showed that in the chick brachial spinal cord there is a larger number of excitatory interneurons crossing the midline compared to the lumbosacral segments, and this is related to changes in the structure of a spinal midline protein that in mammals instructs the wiring that enables limb alternation, ephrin-B3. This is similar to the phenotype of ephrin-B3 and ephrin-A4 null mice which present rabbit-like hopping gait (Kullander et al. 2001, 2003; Butt et al. 2005; Akay et al. 2006; Borgius et al. 2014).

Here we found two pieces of evidence that show that even at early stages, the brachial and lumbosacral spinal cord segment circuits differ. First, we found that in the intact spinal cord both segments produce coordinated rhythmic outputs with burst of the same duration and frequency (Figs. 1, 2, 3), but when separated, and under the same conditions, the brachial spinal cord produces a slower motor rhythm, with longer bursts (Fig. 3 I). While is no possible to deduce why the brachial segments produce a slower rhythm than the lumbosacral, it does suggest that the CPGs in these two different areas of the spinal cord have differences in either the circuit wiring or expression of different receptors. In mammals, different glutamate receptors have been shown to regulate the speed of the locomotor-like activity (Talpalar and Kiehn 2010), thus it is possible that the brachial and lumbosacral spinal cord of chicks differ in their composition of glutamate receptors. Alternatively, our own results show that different concentrations of 5-HT can influence the duration and frequency of bursts (Fig. 2d, e) and therefore it is also possible that the two regions of the chick spinal cord differ in the amount and type of 5-HT receptors. Serotonin has been shown to control frequency and other aspects of locomotion in other vertebrates, including lampreys and several species of mammals (Harris-Warrick and Cohen 1985; Schmidt and Jordan 2000; Sławińska and Jordan 2019).

Second, we found that the brachial and lumbosacral rhythmic outputs are affected differentially by antagonists of inhibitory neurotransmitter, picrotoxin and strychnine (Figs. 4, 5). Both of these antagonist result in a disruption of the rhythmic activity in the lumbosacral spinal cord at lower concentrations than the brachial spinal cord (Figs. 4, 5). Further, strychnine has no effect on the rhythmic output of the brachial spinal cord, while completely supressing it in the lumbosacral (Fig. 5), again suggesting that the brachial and lumbosacral regions have different CPGs. While picrotoxin was originally considered a non-competitive GABA receptor antagonist (Robbins and Van Der Kloot 1958; Takeuchi and Takeuchi 1969), several studies have now shown that it can also inhibits glycine receptors (Wang and Slaughter 2005; Wang et al. 2006). Particularly, picrotoxin has a stronger effect as an antagonist in glycine receptors that contain a α2 subunits (Wang and Slaughter 2005; Li and Slaughter 2007). The chick lumbosacral spinal cord white matter has been shown to express glycine receptors with a α2 subunit at E13 (Harvey et al. 2000), which suggest that the effect we found of picrotoxin in the rhythmic output is, at least partially, mediated by glycine receptors. Consistent with this, the effects of picrotoxin and strychnine, a glycine receptor antagonist (Curtis et al. 1971; Young and Snyder 1973) are very similar (Figs. 4, 5). The disappearance of a rhythmic output in the lumbosacral spinal when glycine receptors are blocked (Fig. 5) shows that even when producing coordinated motor outputs between left and right, the rhythmicity of the motor output in the lumbosacral spinal is glycine dependent, while that of the brachial segments is glycine independent (Fig. 5). This would suggest that the brachial and lumbosacral spinal cord of the chick differ in their glycinergic circuity or receptors. This is not be surprising as glycine and glycinergic interneurons have been shown to be an important part of the left and right alternation circuit in several vertebrates (Droge and Tao 1993; Hagevik and McClellan 1994; Kremer and Lev-Tov 1997). This effect of glycine in the alternation of left and right is at least partially mediated by a group of commissural glycinergic interneurons which have been found in most vertebrates (Gosgnach et al. 2017). In mice, deletion of this population of neurons results in coordination between the left and right fictive locomotion and a hooping phenotype (Talpalar et al. 2013). While these glycinergic interneurons are clearly involved in the alternation of left and right motor outputs, other neuronal populations, including excitatory commissural interneurons are also involved in the alternation of left and right locomotor outputs, and therefore, left–right alteration ultimately depends on a balance of excitatory and inhibitory inputs (Restrepo et al. 2011). As mentioned above, Haimson et al., (2021) has recently show that the brachial segments of the avian spinal cord have an increased number of excitatory interneurons that project to the contralateral side. Our results suggest that the differences in the excitatory/inhibitory balance between the brachial and lumbosacral segments may not be solely due to an increase in excitatory inputs but also a reduction of inhibitory inputs, specifically glycinergic inputs.

### Bilateral coordination

Despite testing several different concentrations of NMDA, 5-HT and dopamine, and combinations thereof, (Figs. 1, 2, 3), we were not able to produce an alternating rhythmic output in the lumbosacral spinal cord of the chick. It is possible that at this embryonic stage (E11–E14) the chick spinal cord is not able to produce alternating locomotor patterns. Previous studies have shown that the chick spinal cord from about E10 to E14 can produce alternation of antagonist muscles on the same side (Barry and O’Donovan 1987; Ho and O’Donovan 1993; Sholomenko and O’Donovan 1995), which we observed as well (Fig. 4a, b). In chick embryos older than E15 (but not before), stimulation of the brainstem locomotor centres can evoke locomotor activity, including both simultaneous and alternating leg movements, as well as wing flapping (Valenzuela et al. 1990), which could suggest that the alternating circuit is not completed until E15. Unfortunately, the size of the spinal cord in E15 embryos makes it impossible to survive in an in vitro preparation. In mice, the left–right alternating circuit also seems to develop later, as early mice embryos (E15) also produce only synchronous rhythmic patterns (Branchereau et al. 2000). One possibility would be that in the whole spinal cord, the brachial segments, which should produce left/right synchronous outputs are driving the lumbosacral segments. Our results show not only that the isolated lumbosacral segments produce a coordinated output in the absence of connections with the brachial segments (Figs. 3, 5), but suggest that the lumbosacral segments are driving the rhythmic brachial output. First, we found, under a variety of conditions, that the brachial output always lags behind that of the lumbosacral output (Figs. 2b, f, g, 3e, 4e–g, 5a–c). This has been reported previously in a chick in vitro preparation (Ho and O’Donovan 1993). Second, we found that after separating the brachial segments from the lumbosacral segments, the burst duration and burst frequency of the brachial segments was significantly slower than the burst frequency of the brachial segments when they were still connected to the rest of the spinal cord, while that the burst frequency of the lumbosacral segments did not change after loosing its connection to the brachial segments. (Fig. 3c–i). This shows that the burst characteristic in the whole spinal cord is closer to that of the isolated lumbosacral than the brachial segments. Finally, when either strychnine or picrotoxin was added to the whole spinal cord, the leg rhythm was disrupted first followed by the brachial segments (Figs. 4e–h, 5), even though the brachial segments were not affected by the blocking of glycine when not attached to the lumbosacral segments, suggesting that disruption of the lumbosacral rhythm eventually propagates to the brachial segments. The complete coordination between the two CPGs suggests that there are strong connections between the rhythmic centers at this embryonic stage, particularly from the lumbosacral to the brachial ones.

Alternatively, the lack in alternating movements in the lumbosacral spinal cord of chick could also reflect spinal circuits that are not only able to produce post-hatching behaviors, like walking and flying, but also behaviors present before hatching, particularly hatching behavior. To be able to exit the eggs, chicks perform a series of coordinated movements, including kicking of their legs together in a rhythmic fashion (Bekoff and Kauer 1982, 1984). Chick embryos show movement as early as E4 (Hamburger 1968; Bekoff 1976), and while this movements are highly variable and not always coordinated between the two legs or between the legs and the wings (Provine 1980; Watson and Bekoff 1990; Chambers et al. 1995), their motor patterns resemble the motor patterns of both hatching and walking (Watson and Bekoff 1990; Bekoff 1992, 1995). Therefore, is possible that the lumbosacral CPGs in chicks develop the capacity of producing both coordinated and alternating leg movements, and that in the absence of central control they default to the motor pattern that occurs earlier in development, like kicking with the legs.

## Conclusion

In this study we provide evidence that the spinal cord of bird’s harbors two distinct types of CPGs that generate two different motor patterns: synchronous wing beating with the forelimbs and asynchronous hind limbs movements for walking. Interestingly, it is likely that the evolution of flight and bipedalism in birds have resulted in even more independent locomotor modules and CPGs. For instance, when flying, some birds move their tails rhythmically in coordination with the wings, particularly during take off and landing (Gatesy and Dial 1996; Gatesy and Baier 2005). Additionally, when walking, many birds show a rhythmic forward and backward movement of their heads, “head-bobbing”, and these head movements are, in some species, synchronized with the leg movements (Necker 2007; Hancock et al. 2014). Thus, it is possible that both neck and tail CPGs exist in the spinal cord of some birds, and that these coordinate with the output of the locomotor CPGs.

It would be interesting to know if further differences in circuitry exist among birds with divergent flight patterns. For example, one would expect that the CPG producing the slow wing beat of a large heron to be different to that of a hummingbird beating the wings rapidly. Beyond birds, other animals have evolved locomotor patterns that require synchronous movements of the limbs. This includes flying in bats, but also hopping in a series of mammals, including rabbits, some rodents and kangaroos (McGowan and Collins 2018). Furthermore, many small birds, especially passerines, hop rather than walk, although this may not be as obligatory as the coordinated movement of the wings when flying (Provini and Höfling 2020). Nonetheless, it is likely that the CPG of those species have evolve to produce species specific locomotor pattern, but given the diversity and independent origins of this similar locomotor patterns, that no all have come up with the same solution, providing a rich diversity in which to look for common and divergent circuits designs (Katz and Harris-Warrick 1999; Katz 2016).

## Data Availability

The data that support the findings of this study are available on request from the corresponding author, CGI.
